# Camera–Radar Fusion with Modality Interaction and Radar Gaussian Expansion for 3D Object Detection

**DOI:** 10.34133/cbsystems.0079

**Published:** 2024-01-30

**Authors:** Xiang Liu, Zhenglin Li, Yang Zhou, Yan Peng, Jun Luo

**Affiliations:** ^1^ Institute of Artificial Intelligence, Shanghai University, Shanghai, China.; ^2^ School of Future Technology, Shanghai University, Shanghai, China.; ^3^ State Key Laboratory of Mechanical Transmission, Chongqing University, Chongqing, China.

## Abstract

The fusion of millimeter-wave radar and camera modalities is crucial for improving the accuracy and completeness of 3-dimensional (3D) object detection. Most existing methods extract features from each modality separately and conduct fusion with specifically designed modules, potentially resulting in information loss during modality transformation. To address this issue, we propose a novel framework for 3D object detection that iteratively updates radar and camera features through an interaction module. This module serves a dual purpose by facilitating the fusion of multi-modal data while preserving the original features. Specifically, radar and image features are sampled and aggregated with a set of sparse 3D object queries, while retaining the integrity of the original radar features to prevent information loss. Additionally, an innovative radar augmentation technique named Radar Gaussian Expansion is proposed. This module allocates radar measurements within each voxel to neighboring ones as a Gaussian distribution, reducing association errors during projection and enhancing detection accuracy. Our proposed framework offers a comprehensive solution to the fusion of radar and camera data, ultimately leading to heightened accuracy and completeness in 3D object detection processes. On the nuScenes test benchmark, our camera–radar fusion method achieves state-of-the-art 3D object detection results with a 41.6% mean average precision and 52.5% nuScenes detection score.

## Introduction

Three-dimensional (3D) object detection plays a pivotal role in autonomous driving, leveraging sensor data to discern the position, size, and category of objects within a 3D world coordinate system. This process ensures accurate estimation of the surrounding environment, providing reliable observational outcomes crucial for prediction and planning. In the context of autonomous driving, erroneous recognition has the potential to profoundly influence subsequent trajectory prediction and path planning, thereby compromising the safety and reliability of autonomous driving systems.

Presently, prevailing research in 3D object detection relies on RGB images provided by camera sensors. Cameras offer rich visual information and possess significant advantages in recognizing object categories. However, the object is projected onto the image with fewer pixels at long distances, causing details and textures to blur. This reduction in image resolution poses challenges for the object detection algorithm, hindering precise feature extraction and depth estimation. Additionally, under low-light conditions, background noise intensifies in the image, diminishing the contrast between the target and its surroundings. Consequently, the boundaries of the targets become indistinct, complicating the object detection algorithm’s ability to differentiate between the target and the ambient environment. In such scenarios, the detection performance of 3D object detection is often compromised [1,2].

In autonomous driving, various sensors are equipped alongside cameras, including LiDARs and radars. LiDAR offers precise distance measurements and enables accurate detection of target object shapes. However, its widespread utilization in large-scale commercial applications is limited due to its higher cost than other sensors. Additionally, LiDAR exhibits weaker color and pattern recognition capabilities when contrasted with camera sensors. On the other hand, radar excels in sensing speed and distance, rendering it suitable for detecting moving targets. Moreover, due to its robust penetration capabilities, radar is less impacted by adverse weather conditions such as rain and snow. Nonetheless, its limitations are offering sparse point cloud information about object shapes and possessing lower resolution, which restricts its ability to provide the same level of rich semantic information as camera sensors [3,4]. Therefore, the fusion of multiple sensors for 3D object detection has emerged as a new trend and direction. This approach allows for the integration of the strengths of each sensor, compensating for their individual limitations.

Hence, based on the rapid advancements in 3D object detection algorithms, this paper researches a fusion method integrating camera and radar sensors to enhance the accuracy of 3D object detection.

The fusion of camera and radar sensors can improve the accuracy of 3D object detection but also faces several challenges: (a) Large differences in modalities: Sensors provide data in varying formats; the camera offers RGB images, whereas radar produces 3D point clouds. Moreover, sensors operate within distinct coordinate systems:perspective view (PV) for cameras, and bird’s-eye view (BEV) for radars. Fusing sensor data with different representations and coordinate systems is a complex task. (b) Inaccurate radar height measurement: Radar’s operational principles and limitations may introduce errors in the height measurement of 3D point clouds. This inaccuracy results in imprecise or missing height information for objects, posing a challenge in achieving reliable detection. (c) Sparse radar point clouds: Radar data often exhibit sparsity compared with cameras. Numerous objects visible to cameras may yield only a few radar reflections, posing significant challenges for camera–radar signal association and fusion. Addressing these challenges demands meticulous handling of image and radar data, employing appropriate fusion methodologies tailored to the unique features of each modality, and ultimately achieving accurate 3D object detection.

In existing research, certain scholars have opted to project radar point clouds onto images [5,6] or transform image features into BEV features, subsequently performing fusion at BEV [7–9]. However, projecting radar points onto images results in a notable loss of geometric information. Back-projecting camera features onto sparse radar points leads to a significant loss of semantic information [10]. These methods, unfortunately, only partially preserve the information provided by both sensors throughout the fusion process. Hence, researchers need to explore more effective ways to integrate image and radar features. This exploration is essential for taking full advantage of each sensor, minimizing information loss during fusion, and ultimately enhancing detection accuracy.

The paper proposes a query-based modality interaction framework with a radar expansion for camera and radar fusion in 3D object detection. The framework employs queries to sample and aggregate features from different sensors, continuously enrich and refine relevant features for 3D object detection within the queries, and achieve accurate predictions. A key innovation of this framework lies in introducing an interaction module between image and radar features, facilitating information exchange while preserving the unique advantages of each modality. Regarding radar data processing, an ingenious application of the Gaussian function is utilized to expand radar voxel features, enhancing the accuracy and robustness of 3D object detection.

The main contributions of our paper are summarized as follows:

1. A camera–radar fusion framework for 3D object detection is designed, which employs 3D queries to sample and aggregate features from both cameras and radars through a modality interaction module.

2. A new representation format of radar point cloud, called radar Gaussian expansion module, is proposed, which extends the radar data and exhibits excellent performance.

3. The proposed model is evaluated on the challenging benchmark dataset nuScenes with extensive experiments. Our model achieves promising performance (41.6% mean average precision [mAP] and 52.5% nuScenes detection score [NDS]) in camera–radar multi-modal 3D object detection.

### Related work

#### Camera-based 3D detection

3D object detection requires simultaneously obtaining category labels and bounding boxes for all predefined objects. Inspired by the 2D object detection method FCOS (fully convolutional one-stage object detector) [11], FCOS3D (fully convolutional one-stage 3D object detector) [12] decouples 3D properties into 2.5D (position offset + depth) and 3D properties (size, rotation angle, etc.), treating it as a 2D object detection problem. However, this approach results in many overlapping boxes, leading to subpar performance. To address this challenge, Probabilistic and Geometric Depth (PGD) [13] leverages probabilistic depth uncertainty and the geometric relationships between coexisting objects, ensuring accurate depth estimation and mitigating the inaccurate depth predictions encountered in FCOS3D. While this enhancement significantly improves accuracy, it comes at the expense of increased computational costs and inference delays.

Query-based 3D object detection is an end-to-end approach employing transformers [14]. DEtection TRansformer 3D (DETR3D) [15] improves upon DEtection TRansformer (DETR) [16] for 3D object detection by projecting learnable 3D queries into the image, sampling relevant features, refining the queries using attention mechanisms, and not requiring Non-maximum Suppression (NMS) processing due to the presence of a set prediction module. However, the continuous projection and sampling operations in the DETR3D model hinder its practical deployment. Position Embedding Transformation (PETR) [17] improves this process by incorporating 3D position encoding into image features and establishing positional relationships across different viewpoints in 3D space. Nonetheless, uncertainties persist in the depth information of image features within the 3D positional encoding, impacting prediction accuracy. Utilizing precise depth information from LiDAR or radar sources would enhance the model’s performance.

Moreover, a novel approach involves transforming perspective-view image features into BEV features and utilizing the spatial distribution of features in 3D space for object detection. Bird’s-eye view detection (BEVDet) [18] achieves this by converting perspective-view image features into BEV features, implementing customized data augmentation strategies, and employing enhanced NMS methods, striking an optimal balance between accuracy and efficiency. BEVDet4D [19] extends the concept further by preserving BEV features from previous frames, aligning and concatenating them with current frame features, and utilizing queries on two candidate features to obtain temporal clues, compensating for errors in speed estimation derived from single-frame images. Similarly, BEVFormer [17] employs multi-head attention to perform view transformation on neighboring frames’ images, allowing flexible fusion of time and space features. Notably, accurate depth information is crucial for BEV features and is typically derived from the depth probability distribution. As the detection range widens, the precision of depth estimation becomes increasingly vital.

The camera-only model exhibits commendable performance but lacks sufficient depth information, posing challenges in accurately predicting the bounding boxes of 3D objects. Numerous methods use geometric constraints and shape priors to deduce depth information from images, offering a promising path for refining 3D object localization. However, compared to camera–radar fusion models, a substantial disparity persists in their performance levels.

#### Camera–radar 3D detection

The fusion of camera and radar aims at leveraging the strengths of a diverse sensor combination to achieve accurate 3D object detection. According to different fusion methods, they can be divided into 3 groups:

**Based on radar-to-image projection**. This method involves the projection of radar point clouds onto the RGB image plane, creating sparse radar images for subsequent feature extraction and fusion. Approaches such as [20–23] project radar point clouds onto the vertical plane of the original camera image, incorporating radar-specific details like distance and velocity as additional channels. Rather than utilizing raw radar data directly, Nabati and Qi [24,25] project radar detection results, instead of radar point clouds, onto the image plane to define areas of interest within the image. A distinct approach described in [5] generates a radar point density map (RPDM) using radar velocity and energy, resulting in feature maps with enhanced gradient features. This method enables more effective learning through neural networks. The CenterFusion technique [6] introduces frustums in the image based on initial camera detections. These frustums are instrumental in associating relevant radar points with camera features and filtering out noise, significantly enhancing detection performance. Conversely, RadSegNet [26] encodes semantic information from the image and incorporates it into radar point clouds, facilitating subsequent feature extraction.

Frameworks of this kind primarily consider radar point clouds as supplementary channels of the image, applying image processing techniques to handle radar information. The primary challenge arises from the absence of explicit height information in radar data. Conventionally, candidate areas corresponding to radar points projected onto the image are depicted using pillars or lines to indicate height. However, this approach might lead to incorrect associations between radar data and unrelated image regions, thereby compromising the accuracy of the correlation between images and radar points.

**Methods based on BEV features.** The camera provides a perspective view RGB image, while the radar provides point clouds in BEV. Converting camera features from a perspective view to BEV and fusing them with radar features proves to be a robust strategy. This approach has proven superior in the camera–LiDAR fusion model BEVFusion [10,27], unifying multi-modal features in the shared BEV representation space. Within the camera–radar fusion framework, RC-BEVFusion [7] concatenates BEV features generated from images with radar features for subsequent fusion. Existing research indicates that developing more reliable image BEV features significantly enhances the accuracy of detection models [18,28–30]. RCM-fusion [9] introduces an instance-level fusion module to augment BEV feature fusion. Notably, in [7,9], the generation of image and radar features operates independently. In [8], radar point cloud supplements depth information during BEV feature generation. Another approach, Camera Radar Net (CRN) [31], employs a cross-attention mechanism between image BEV features and radar BEV features. This attention-based method demonstrates a superior ability to correlate and fuse multi-modal features compared to simple concatenation.

**Based on proposals.** Methods in this category aim to fuse information from diverse sensors by combining object proposals. RCM-fusion [9] leverages radar data to guide the transformation of image features into BEV features, conducts feature-level fusion, and subsequently generates 3D bounding boxes. It further employs grid-based proposal feature fusion for instance-level refinement. Building upon the success of DETR3D [15] in 3D object detection from images, many sensor fusion models have embraced similar strategies. TransCAR [32] initiates queries from images, learns radar features from multiple accumulated radar scans, and utilizes transformer decoders to capture interactions between radar features and visual update queries. Similarly, FUTR3D [33] extracts features from images, LiDAR, and radar to initialize queries and employs transformers to update each query. Furthermore, CRAFT [34] uses a cross-attention mechanism to fuse proposals generated from images and radar.

## Materials and Methods

Our framework mainly comprises the following modules: (a) Camera and Radar Processing: This section includes the radar Gaussian expansion module, which expands radar data within voxels using Gaussian distribution to minimize projection errors. Additionally, it encompasses feature extraction procedures for both image and radar modalities. (b) Modality Interaction Module: The modality interaction module integrates radar features into image features, enhancing the overall quality of image features. (c) Feature Sampling and Update Module: In this module, all queries utilized for 3D object detection are iteratively updated. The module performs feature sampling on image and radar features based on the location of query reference points and aggregates these features to refine and update the queries. By employing a transformer [14] decoder for updates, all queries can be iteratively enhanced, enabling the prediction of 3D bounding boxes based on these refined queries. The paper proposes a query-based modality interaction framework with a radar expansion for camera and radar fusion in 3D object detection.

**Fig. 1:**
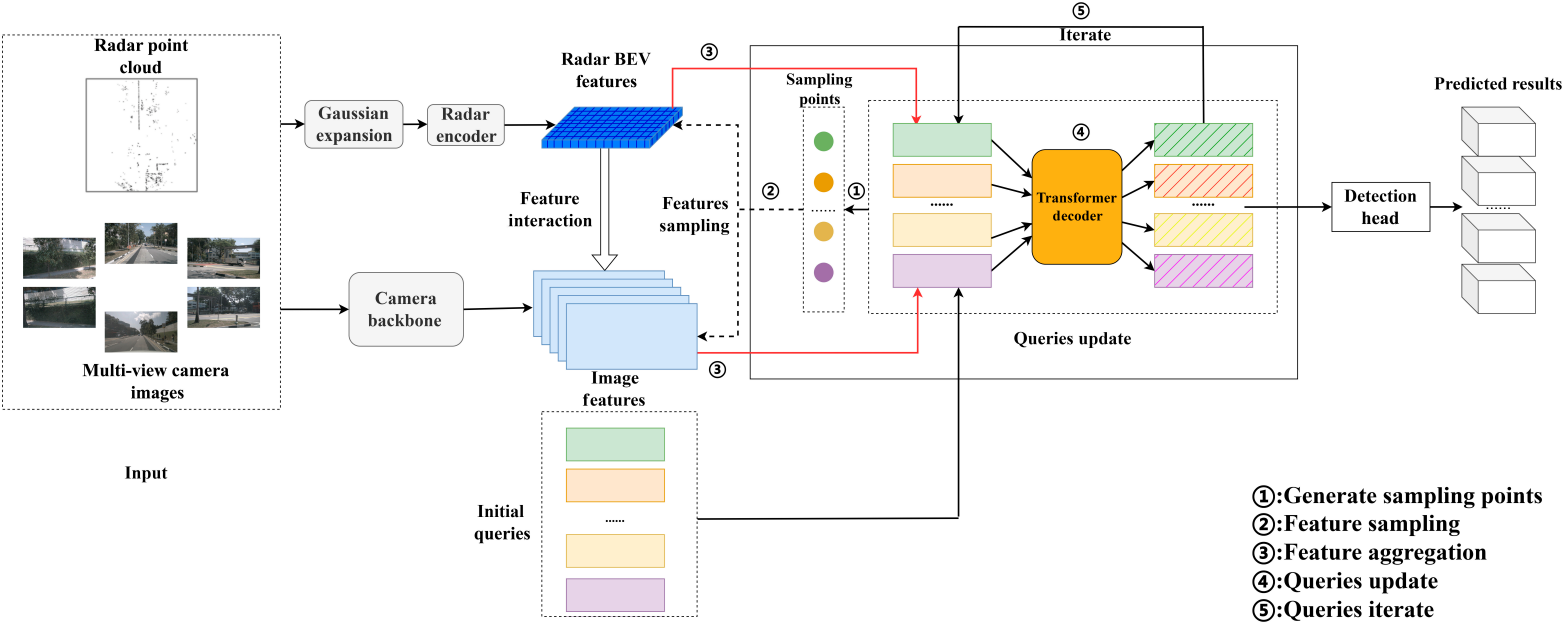
. Overview of the model framework. The image and radar data are separately encoded, and the radar features are fused with the image features. Each query extracts features from images and radar based on 3D reference points, and aggregates features from different modalities. Finally, a transformer decoder is used to predict 3D bounding boxes from the queries.

In this paper, synchronization and fusion of different sensors are achieved through a projection-based approach. Firstly, in the Modality Interaction Module, image features are associated with radar voxels through back-projection. Secondly, in the query aggregation stage, reference points generated by queries are distributed and projected onto the features of different sensors to achieve synchronization of different sensor features. An overview of our method is shown in Fig. 1.

### Camera and radar processing

**Fig. 2:**
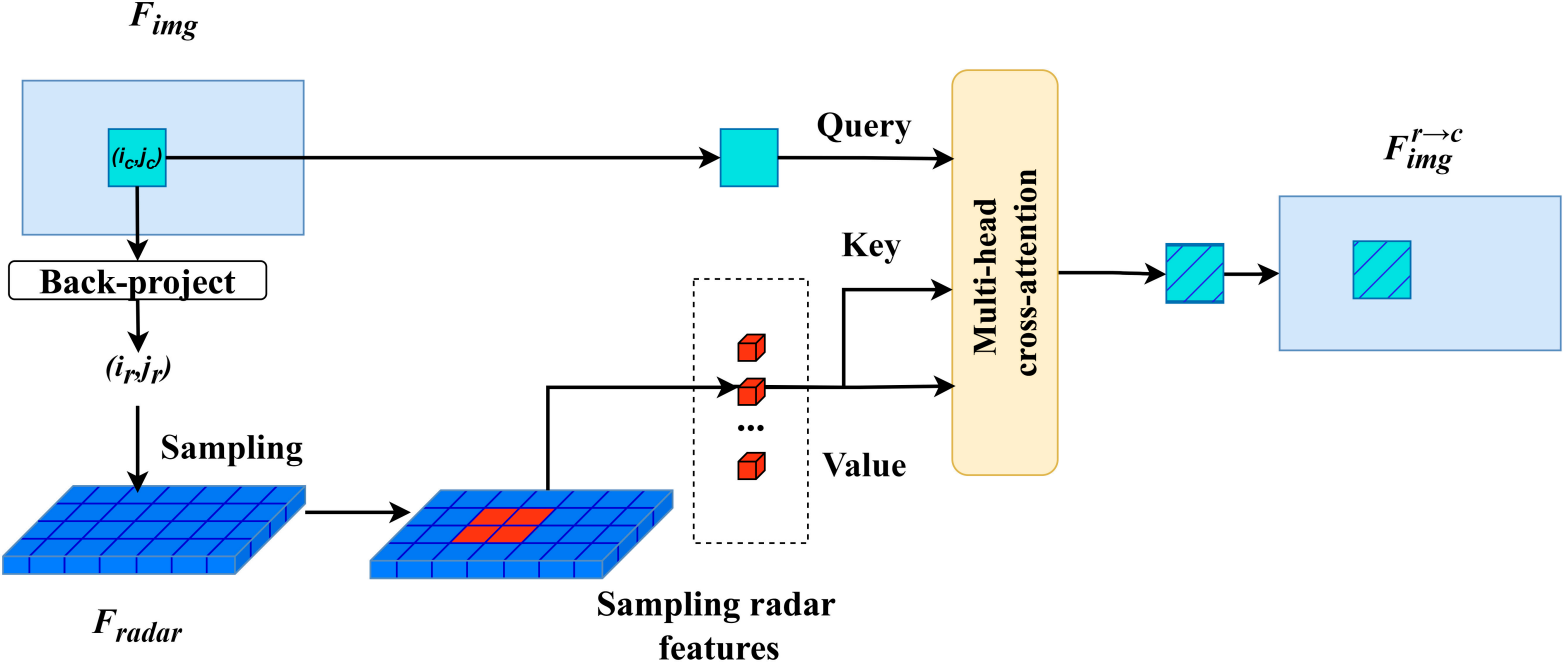
. An overview of the radar Gaussian expansion module. For a specific radar voxel, serving as the central voxel, the radar data within this voxel are weighted based on the positions of the surrounding voxels using Gaussian distribution. Subsequently, this weighted radar information is allocated to the surrounding voxels.

**Radar Gaussian expansion module.** For radar sensor data, transforming the original discrete point cloud data into a voxel-based representation reduces data complexity and simplifies the processing workflow. Voxelized point cloud data suit various information processing and computation tasks. Furthermore, a novel radar voxel representation method is designed and applied in the radar processing stage to mitigate the influence of projection errors during reference point projection and sampling. By using the position and velocity of radar points as input channels and voxelizing the radar point cloud, radar voxels *V*(*u*) are obtained. The radar Gaussian expansion module is applied to the voxelized radar points. For all radar points within a voxel, Gaussian distribution is used to scatter the data of radar points into surrounding voxels. Subsequently, feature extraction is conducted on the voxelized radar point cloud, as follows:

$$V\left(u_{i}\right)=\sum_{j}‍G_{\sigma_{0}}\left(u_{i}\right)\cdot V\left(u_{\mathrm{ij}}\right)$$ (1)

where *V* represents the voxel space. *u_i_* denotes the*i*-th voxel of the radar, while *u_ij_* refers to the voxel itself and the neighboring voxels. Each voxel accumulates the radar data allocated from the surrounding voxels. $G_{\sigma_{0}}$ is the Gaussian kernel with standard deviation *σ*_0_. In this way, the fixed positions of radar points are transformed into probabilistic positions, and the discrete and sparse radar voxels are converted into locally continuous and dense radar voxels. An overview of the radar Gaussian expansion module is shown in Fig. 2.

**Fig. 3:**
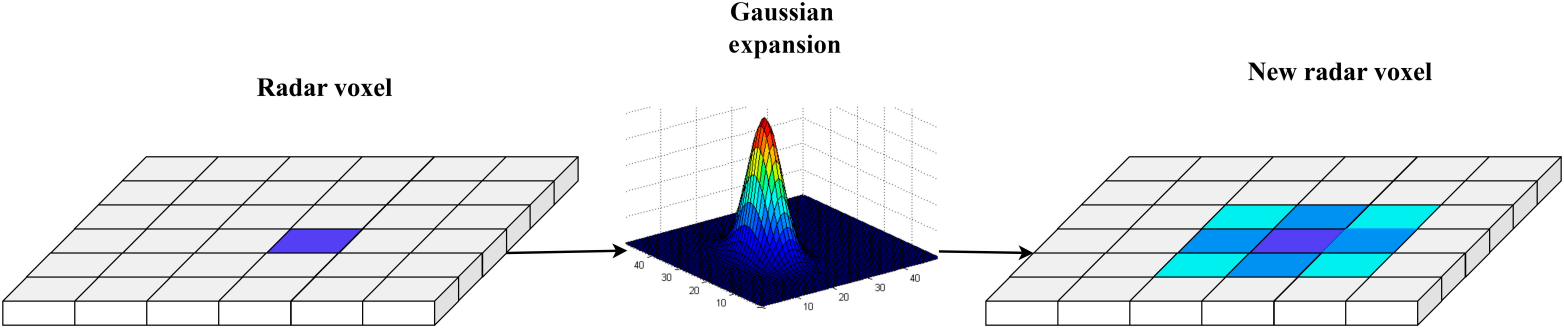
. Overview of radar-to-camera feature fusion. Back-project the image feature at position (*i_c_*, *j_c_*) in the image feature and locate the corresponding position (*i_r_*, *j_r_*) in the radar feature. Retrieve the nearest several radar features to position (*i_r_*, *j_r_*), and represent them in the image as red cubes. The image feature acts as a query, while the radar feature acts as key and value. Feature fusion is achieved through cross-attention learning.

**Image and radar feature extraction.** In our model framework, we utilize surrounding camera images in autonomous driving. ResNet [35] and Feature Pyramid Network (FPN) [36] are employed for image feature extraction, which outputs multi-scale features for each image. The $F_{\mathrm{img}}^{i}\in R^{C\times H\times W}$ denotes multi-scale vision features of the *i*-th camera.

In radar processing, feature extraction is conducted on the radar voxels processed by the radar Gaussian expansion module. A multi-layer perceptron (MLP) network *MLP_rad_* is employed to extract features for each radar voxel, denoted as *F_radar_*:

$$F_{\text{radar}}=MLP{\;}_{\mathrm{rad}}\left(V\left(u\right)\right)$$ (2)

### Modality interaction module

Conventional fusion methods typically aggregate multiple modal inputs into a hybrid feature map to represent the fusion feature in a single-modal form. However, this approach often leads to information loss. The modality interaction module, in contrast, is designed to integrate multiple modalities while preserving the original information.

The modality interaction module is crucial in enhancing image features through camera–radar fusion. Images provide rich semantic information, while radar offers more precise position and velocity data. The modality interaction module effectively combines features from both the image and radar modalities. It updates the image features while retaining the original radar features to prevent any loss of radar information during the fusion process. Therefore, the modality interaction module takes modality-specific features extracted independently by the image backbone and radar backbone as inputs. It associates the radar features with the image features using the intrinsic and extrinsic parameters of the camera. Cross-attention learning is employed to fuse image and radar features, obtaining improved image features while keeping the original radar features unchanged. These improved image and radar features are then jointly fed into the subsequent stages of the model. Following [37], the detailed process is as follows:

1. **Radar-to-camera feature fusion**

To enable the fusion of radar and image features, it is essential to establish a mapping between camera coordinates *c* and radar coordinates *r*. This mapping allows us to identify the corresponding radar features for each feature in the image, denoted as *Mc-r*. Specifically, leveraging the intrinsic and extrinsic parameters of the camera, each radar point in radar space is projected onto multiple camera images. The depth information radar provides is utilized to create a sparse depth map. For image pixels lacking radar point projections, depth completion [38] is applied to assign depth information, generating a dense depth map. By utilizing this dense depth map, each pixel (*i_c_, j_c_*) in the image is back-projected into the radar point cloud and associated with the nearest radar points, establishing the mapping relationship *Mc-r* between image pixel coordinate (*i_c_, j_c_*) and radar point coordinate (*i_r_, j_r_*).

In terms of feature fusion, the image feature at position (*i_c_, j_c_*) serves as the query ($q=F_{\mathrm{img}}^{\left(i_{c},j_{c}\right)}$), while the back-projected associated radar features $F_{\text{radar}}^{M_{c-r}(i_{c},j_{c})}$ act as the key (*k*) and value (*v*). Cross-attention learning subsequently fuses these features, as shown in Fig. 3.

$$F_{\mathrm{img}}^{r\to c}=g^{r\to c}(F_{img},F_{radar})$$ (3)

$$\begin{array}{c} g^{r\to c}\left(F_{\mathrm{img}},F_{\text{radar}}\right)=\sum_{k,v\in F_{\text{radar}}}‍\text{softmax}\left(\frac{\mathrm{qk}}{\sqrt{d}}\right)v \end{array}$$ (4)

where $F_{\mathrm{img}}^{r\to c}$ represents the image feature after fusing the radar features, and *g*^*r* → *c*^(·) is the method of fusing the two modalities through attention mechanism. *d* denotes the channel number of image features.

2. **Local attention among image features**

The local attention mechanism is incorporated to facilitate modality interaction, enhancing the model’s capability to concentrate on the target region for extracting relevant features. For every position within the image feature, a query (*q*) is generated to represent its features, while the key (*k*) and value (*v*) are generated for adjacent positions, representing neighboring features. The features are denoted as $F_{\mathrm{img}}^{c\to c}$.

$$F_{\mathrm{img}}^{c\to c}=g^{c\to c}(F_{img},F_{img})$$ (5)

$$\begin{array}{c} g^{c\to c}(F_{img},F_{img})=\sum_{k,v\in F_{\mathrm{img}}}‍\;\text{softmax}\left(\frac{\mathrm{qk}}{\sqrt{d}}\right)v \end{array}$$ (6)

3. **Aggregation of image features**

Then, the image features ($F_{\mathrm{img}}^{r\to c}$ and $F_{\mathrm{img}}^{c\to c}$) derived from the aforementioned process are introduced into a neural network alongside the original image features (*F_img_*), facilitating the aggregation of these features and generating the definitive improved image features output.

$$\begin{array}{c} F_{\mathrm{img}}^{\prime }=MLP{\;}_{\mathrm{agg}}\left(\;\text{Concat}\left({\mathrm{MLP}\;}_{\mathrm{agg}}\left(\;\text{Concat}\left({\text{F}}_{img}^{c\to c},F_{img}^{r\to c}\right)\;\right),{\text{F}}_{\mathrm{img}}\right)\;\right) \end{array}$$ (7)

where *Concat* denotes element-wise concatenation and *MLP_agg_* is the neural network used to aggregate image features.

### Feature sampling and update module

This module takes a set of object queries $Q={\left\{q_{i}\right\}}_{i=1}^{N_{q}}$, aggregated image features $F_{\mathrm{img}}^{\prime }$, and radar features *F_radar_* as inputs to generate new queries *Q*′. Predict 3D bounding boxes and categorial labels from the new queries [15,16,33].

Before sampling features, it needs to determine the position of the query in 3D space. The 3D position $c_{i}^{3D}$ of each query *q_i_* is obtained by encoding the query using *MLP_ref _*, as follows:

$$c_{i}^{3D}=MLP{\;}_{ref}\left(q_{i}\right)$$ (8)

where $c_{i}^{3D}$ represents the position of the query *q_i_* in 3D space and is referred to as a reference point.

Due to differing perspectives between cameras and radar, distinct methods are utilized to obtain projected points for images and radar features. For image features, reference points are projected onto features from different receptive fields of multi-view images. Utilizing the intrinsic and extrinsic parameters of the camera, the projected positions of reference points $c_{i}^{3D}$ on the image are obtained, representing the projected points (or sampling points) $c_{\mathrm{im}}^{\mathrm{PV}}$:

$$c_{\mathrm{im}}^{\mathrm{PV}}=T_{m}c_{i},m=1,\cdots ,6$$ (9)

where *m* is the *m*-th camera image and *T_m_* denotes the matrix transforming reference points onto multi-view images.

The image features from multi-view images at different scales are sampled and weighted-summed to obtain the ultimate image features, as follows:

$$f_{\mathrm{img}}^{i}=\sum_{j}‍\sum_{m}‍\sigma_{\mathrm{img}}^{\mathrm{ijm}}\cdot F_{\mathrm{img}}^{\mathrm{jm}}\left(c_{\mathrm{im}}^{\mathrm{PV}}\right)$$ (10)

where $f_{\mathrm{img}}^{i}$ denotes the sampling feature of the *i*-th query, $F_{\mathrm{img}}^{\mathrm{jm}}\left(c_{\mathrm{im}}^{\mathrm{PV}}\right)$ represents a feature bilinearly sampled from the *j*-th scale feature map of the *m*-th image at the sampling point $c_{\mathrm{im}}^{\mathrm{PV}}$ for the *i*-th query. The features of this sampling point will be set to zero if the position of the sampling point is outside the image area. $\sigma_{\mathrm{img}}^{\mathrm{ijm}}=MLP{\;}_{img}\left(q_{i}\right)$ signifies a weighting factor obtained from queries using a neural network.

In the radar features, for each query *q_i_*, the *K* nearest radar points $\left\{c_{1}^{r},\;c_{2}^{r},\;c_{3}^{r},\;\cdots ,\;c_{K}^{r}\right\}$ to the reference point $c_{i}^{3D}$ are selected. These *K* radar points are utilized as sampling points for the reference point on the radar feature *F_radar_*. Subsequently, the radar features sampled from the *K* points are weighted and summed up:

$$f_{\text{radar}}^{i}=\sum_{k=1}^{K}‍\sigma_{\text{radar}}^{\mathrm{ik}}\cdot F_{\text{radar}}\left(c_{k}^{r}\right)$$ (11)

where $\sigma_{\text{radar}}^{\mathrm{ik}}=MLP{\;}_{\text{radar}}\left(q_{i}\right)$ is also a weighting factor.

After obtaining the sampling features for each query, we concatenate the features from different modalities and employ an encoder *MLP_fus_* to obtain the fusion features $f_{\mathrm{fus}}^{i}$ for each query, as shown:

$$f_{\mathrm{fus}}^{i}=MLP{\;}_{\mathrm{fus}}\left(\text{Concat}\left({\text{f}}_{img}^{\;i}\text{,}f_{\text{radar}}^{i}\right)\right)$$ (12)

The existing query is refined through the aggregation of features obtained from the query:

$$q_{i}^{\prime }=q_{i}+f_{\mathrm{fus}}^{i}$$ (13)

Then, a self-attention module is embedded to learn the interaction among all 3D object queries, capture global information, and prevent multiple queries from converging onto the same object.

$$Q^{\prime }=Q+\mathrm{SA}\left(Q\right)$$ (14)

where *SA*(·) is self-attention.

Finally, use two MLPs for each query to predict a bounding box and a classification label. Calculate the loss between predictions and ground truths, update the queries based on the loss, and input the queries into the next layer of iteration.

### Loss

The proposed framework employs the set-to-set loss. Let $\hat{Y}=\left(\hat{C},\;\hat{B}\right)$ be predictions and *Y* = (*C*, *B*) be the ground truths, where *C* represents the categorical labels set and *B* represents the bounding boxes set. One-to-one matching between predicted boxes and ground truth boxes is performed through the Hungarian algorithm [39] to solve this assignment problem. Let *α*^∗^ be the optimal assignment function. Then, we calculate the classification loss using focal loss *L_cls_* [40] and the regression loss using L1 loss *L_reg_*. Here, *λ* represents the weights for the classification loss and regression loss, as shown:

$$\begin{array}{c} L\left(Y,\;\hat{Y}\right)=\lambda *L_{\mathrm{cls}}\left(C,\;\alpha^{*}\left(\hat{C}\right)\right)+L_{\mathrm{reg}}\left(B,\;\alpha^{*}\left(\hat{B}\right)\right) \end{array}$$ (15)

## Results

### Experimental setup

**Dataset.** The experiment utilizes the extensively recognized nuScenes dataset [41], the most extensive dataset encompassing multiple sensors and 1,000 distinct scenes. Of these scenes, 700 are designated for training, 150 are designated for validation, and 150 are designated for testing. Each scene includes imagery from 6 cameras, comprising front left, front, front right, back left, back, and back right, in conjunction with 5 radar point clouds. The dataset is meticulously annotated to detect 10 categories: car, truck, bus, trailer, construction vehicle, pedestrian, motorcycle, bicycle, barrier, and traffic cone. The dataset boasts a collection of 1.4 million annotated 3D bounding boxes.

**Metrics.** For the assessment of 3D object detection, our study adheres to the official evaluation metrics provided by nuScenes. The primary metrics in use encompass the mAP and the NDS [41]. Furthermore, the dataset designed several true positive (TP) related metrics to assess different types of errors, including Average Translation Error (ATE), Average Scale Error (ASE), Average Orientation Error (AOE), Average Velocity Error (AVE), and Average Attribute Error (AAE). NDS is computed as a weighted composite of mAP and other attribute metrics, thus comprehensively evaluating the algorithm’s performance across various dimensions.

### Implementation details

In the radar feature extractor, due to the lack of height in radar points, dividing multiple voxels on the *z*-axis is meaningless. The voxel height is set to 8 m, covering the detected range. The voxel size of the radar data is set to [0.8 m, 0.8 m, 8 m]. For RGB image data, take 1,600 × 900 resolution as input and process it using random flipping, random scaling, random rotation, and final cropping. We use MMDetection3D for baseline experiments and implementation of our algorithms. ResNet101 [35] and FPN [36] extract features from multi-view images and obtain 4 different-scale feature maps in this framework. The number of object queries in the transformer decoder is set to 600. The 3D detection head comprises 6 layers, each comprising feature refinement steps and multi-head attention layers. We pre-train the image model for our models, followed by joint training on cameras and radars. Models are trained with AdamW [42] optimizer, in which gradient clip is exploited with a learning rate of 2 × 10^−4^. By default, the total schedule is terminated within 24 epochs with a batch size of 1 per GPU on 2 RTX 3090 GPUs.

### Results and comparison

Our model is compared with other methods, including the camera-only and camera–radar fusion detection models, such as DETR3D, FCOS3D, CenterFusion, and Radiant. Their accuracy on the nuScenes validation set is listed in Table 1. Additionally, testing is conducted on the nuScenes test set, which comprises 150 scenes without ground-truth annotations. The results are presented in Table 2.

**Table 1.** Comparison with state-of-the-art methods on the nuScenes val set. “C” and “R” represent camera and radar, respectively. The best results are bolded and underlined.

Method	Modality	Camera encoder	mAP↑	mATE↓	mASE↓	mAOE↓	mAVE↓	mAAE↓	NDS↑
DETR3D [15]	C	ResNet101	0.346	0.765	0.269	0.392	0.879	0.210	0.422
PETR [17]	C	Vovnet	0.404	0.763	0.271	0.431	0.824	0.204	0.460
PolarFormer [43]	C	ResNet101	0.396	0.700	0.270	0.375	0.839	0.211	0.458
BEVDepth [18]	C	Resnet50	0.359	0.612	0.269	0.507	0.409	0.201	0.480
BEVStereo [28]	C	Resnet50	0.372	**0.598**	0.270	0.438	**0.367**	0.190	0.500
FCOS3D [12]	C	ResNet101	0.321	0.754	**0.260**	0.486	1.332	**0.158**	0.395
PGD [13]	C	ResNet101	0.358	0.667	0.264	0.435	1.276	0.177	0.424
CenterFusion [6]	C+R	CenterNet	0.332	0.649	0.263	0.535	0.540	0.142	0.453
FCOS3D+ RADIANT [44]	C+R	ResNet101	0.363	0.653	0.262	0.499	1.351	1.000	0.340
PGD+ RADIANT [44]	C+R	ResNet101	0.383	0.617	0.265	0.489	1.293	1.000	0.359
FUTR3D [33]	C+R	ResNet101	0.390	0.675	0.274	0.393	0.431	0.197	0.498
Our	C+R	ResNet101	**0.414**	0.625	0.276	**0.365**	0.382	0.178	**0.525**

**Table 2.** Comparison with state-of-the-art methods on the nuScenes test set. The best results are bolded and underlined.

Method	Modality	Camera encoder	mAP↑	mATE↓	mASE↓	mAOE↓	mAVE↓	mAAE↓	NDS↑
CenterFusion [6]	C+R	CenterNet	0.326	0.631	0.261	0.516	0.614		0.449
FCOS3D+ RADIANT [44]	C+R	ResNet101	0.374	0.620	**0.248**	0.452	1.528	1.000	0.355
PGD+ RADIANT [44]	C+R	ResNet101	0.378	0.610	0.256	0.508	1.552	1.000	0.352
FUTR3D [33]	C+R	ResNet101	0.394	0.639	0.266	0.477	0.512	0.126	0.495
Our	C+R	ResNet101		**0.581**	0.263	**0.460**	**0.415**	0.123	

In the nuScenes validation set, our model demonstrates superior performance compared to other models, achieving higher mAP and NDS scores (41.4% and 52.5%, respectively). DETR3D, PETR, and PolarFormer are 3D object detection models based on object queries. These queries are refined through feature sampling and iteratively updated using a Transformer decoder to predict 3D bounding boxes for all queries. Our model exhibits improved accuracy when comparing our results with theirs, with enhancements of 6.8%, 1%, and 1.8% in mAP, respectively. BEVDepth and BEVStereo transform image features into BEV features and conduct 3D object detection from a BEV perspective. Our model outperforms BEVDepth and BEVStereo, increasing mAP by 5.5% and 4.2%, respectively.

Compared to the camera–radar fusion models (CenterFusion, Radiant, and FUTR3D), our model exhibits significant improvements in mAP, with enhancements of 8.2%, 5.1%, 3.1%, and 2.4%, respectively. Our model represents a notable advancement in the realm of camera–radar fusion. Our fusion methodology effectively harnesses the semantic information from images and the geometric information from radar, resulting in enhanced model accuracy.

When considering errors related to translation (ATE), scaling (ASE), orientation (AOE), velocity (AVE), and attribute (AAE), our model demonstrates excellent performance in predicting object direction, translation, and velocity. Additionally, the NDS index of our model surpasses that of the compared models. This achievement can be attributed to our model’s radar Gaussian expansion module, which effectively reduces projection errors, and the modality interaction module, which preserves the original radar features. The position and velocity information provided by radar are utilized effectively.

In the nuScenes test set, we compare our model and other camera–radar fusion models, revealing significant improvements in our model’s performance.

In the performance evaluation based on object categories, the detection outcomes for various object types in our model are presented in Table 3. Among the 10 predefined categories, our model excels in most categories, such as cars, trucks, and buses. Radar exhibits heightened sensitivity toward these metallic objects, and their larger volumes enable them to yield more reliable radar information, which our model effectively utilizes. However, our model faces challenges detecting small objects and non-metallic entities like pedestrians, bicycles, traffic cones, and barriers. These objects generate fewer valid radar returns and prove challenging to differentiate from background clutter, thus impacting the radar’s performance.

**Table 3.** Performance breakdown by object categories. The best results are bolded and underlined.

Method	mAP	Car	Truck	Bus	Trailer	C.V.	Ped	Motor	Bicycle	T.C.	Barrier
PolarFormer [43]	0.396	0.599	0.342	0.418	0.196	0.094	0.460	0.406	0.342	0.588	**0.532**
PETR [17]	0.404	0.588	0.355	0.457	**0.203**	0.098		0.401	**0.375**	0.568	0.502
PGD+ RADIANT [44]	0.383	0.614	0.309	0.382	0.138	0.067	0.462	0.393	0.373		0.484
FUTR3D [33]	0.390	0.631	0.355	0.430	0.176	0.117	0.447	0.421	0.321	0.533	0.468
Our	**0.414**	**0.659**			0.202	**0.138**	0.458	**0.424**	0.326	0.562	0.498

C.V., construction vehicle; Ped, pedestrian; Motor, motorcycle; T.C., traffic cone.

### Ablation studies

In the ablation experiment section, ablation experiments are conducted on the validation dataset to further establish our model’s effectiveness. The impact of different inputs and each module on the model’s performance is analyzed through experiments. This analysis encompasses the improvement brought about by incorporating radar into the original model, the effect of the modality interaction module, the influence of the radar Gaussian expansion module on the model, and the comparison of results obtained with different radar input channels.

**Camera-only and camera–radar.** This section compares the 3D object detection results of the model under the conditions of camera-only and camera–radar fusion, aiming to analyze the improvements brought about by the addition of radar processing to the model. As depicted in Table 4, it is evident that the fusion model exhibits enhancements in various evaluation indicators compared to the single-camera model, leading to an overall improvement in the model’s performance. Experimental evidence conclusively demonstrates that integrating radar data with images enhances the accuracy of 3D object detection. The mAP and NDS exhibit corresponding increases (5.9% mAP, 20.8% NDS).

**Table 4.** Camera-only and camera–radar

Modality	mAP↑	mATE↓	mASE↓	mAOE↓	mAVE↓	mAAE↓	NDS↑
Camera-only	0.355	0.766	0.707	1.556	0.905	0.210	0.317
Camera–radar	0.390	0.674	0.274	0.393	0.431	0.197	0.498
Our	0.414	0.625	0.276	0.365	0.382	0.178	0.525

**Modality interaction module.** In this section, the impact of the modality interaction module on the model is compared, and the differences between bidirectional interaction (camera-to-radar and radar-to-camera) and unidirectional insertion (radar-to-camera) are examined. Bidirectional interaction involves integrating image features into radar features, extending the principle of unidirectional insertion. This leads to enhanced image features and improved radar features.

As one of the well-designed modules of our model, the modality interaction module (unidirectional insertion) has enhanced the model with a 1.1% increase in mAP and a 2.1% increase in NDS. At the same time, we also conduct experiments on bidirectional interaction. However, as an interactive design approach, this method does not yield satisfactory results in our experiments (40.1% vs. 40.5% in mAP). The reason lies in the sparsity of radar points: multiple image pixels can be back-projected onto a single radar voxel, allowing for the aggregation of voxel features. However, when a radar voxel is projected onto an image, it can only capture the features of this pixel, and the enhancement of voxel features is not significant. Therefore, the model shows little improvement even with enhanced radar points using image features, as shown in Table 5.

**Table 5.** Modality interaction module

Modality	mAP↑	mATE↓	mASE↓	mAOE↓	mAVE↓	mAAE↓	NDS↑
Bidirectional	0.401	0.636	0.275	0.385	0.408	0.187	0.519
Unidirectional	0.405	0.636	0.274	0.359	0.476	0.186	0.512
Without interaction	0.390	0.674	0.274	0.393	0.431	0.197	0.498

**Radar Gaussian expansion module.** This section compares the impact of radar voxel features obtained through radar Gaussian expansion with the original radar voxel features on the model. In the experiment, we hope that when expanding the radar voxel, the center voxel still holds a significant weight. Therefore, we set the standard deviation of the Gaussian function to 0.3 (*σ*_0_ = 0.3), with an influence range extending to the 8 surrounding voxels. As demonstrated in Table 6, it is evident that this module has led to improvements (0.9% mAP, 0.13% NDS). The rationale behind this enhancement lies in the radar Gaussian expansion module, which expands the radar data, reduces the influence of projection errors, and minimizes noise as much as possible.

**Table 6.** Radar Gaussian expansion module

Modality	mAP↑	mATE↓	mASE↓	mAOE↓	mAVE↓	mAAE↓	NDS↑
With	0.414	0.625	0.276	0.365	0.382	0.178	0.525
Without	0.405	0.636	0.274	0.359	0.476	0.186	0.512

**Radar input channels.** The dataset has 18 available channels from the radar, encompassing physical quantities such as the position, velocity, reflected intensities, and acceleration of objects, along with their dynamic properties, probabilities of being objects, and probabilities of false alarms. Only the position and velocity data are chosen as inputs for our experiments. We investigate the impact of different radar input channels on the model. As indicated in Table 7, utilizing additional channels does not enhance the model’s performance (40.5% vs. 40.3% in mAP).

**Table 7.** Radar input channels

Modality	mAP↑	mATE↓	mASE↓	mAOE↓	mAVE↓	mAAE↓	NDS↑
Our	0.405	0.636	0.274	0.359	0.476	0.186	0.512
Whole	0.403	0.651	0.275	0.378	0.394	0.178	0.514

## Discussion

In this paper, we propose a camera–radar fusion model for 3D object detection, enhancing existing query-based models by incorporating a radar Gaussian expansion module and a modality interaction module. Its advantages are as follows: (a) The radar Gaussian expansion module expands radar data by converting the fixed positions of radar points into probabilistic positions, transforming discrete radar voxels into more continuous ones, and mitigating projection errors. (b) The modality interaction module facilitates feature fusion between camera and radar data, achieving multi-level fusion of image and radar features: feature fusion and query fusion, preserving the unique characteristics of each modality. These modules collectively enhance the model’s predictive accuracy. Rigorous experiments conducted on the nuScenes benchmark dataset demonstrate the superior performance of our proposed model. It surpasses other camera-only methods and camera–radar fusion models.

Then, we will propose future research work based on the potential limitations of this paper. (a) The parameters of the Gaussian distribution in the radar voxel Gaussian expansion module are empirically set and may not be suitable for the current model. The parameters of the Gaussian distribution have a significant impact on voxel expansion. Future experiments will explore dynamically selecting the parameters of the Gaussian distribution in the model. (b) In the modality interaction module, only the radar features are fused into the image features, and the image features are not fused into the radar features. It has been observed in experiments that the bidirectional interaction effect is not significant. Therefore, in future work, we plan to adjust or replace the fusion method to achieve this bidirectional interaction. (c) During the aggregating of multi-modal sampling features, the current approach concatenates the object query features with the sampling features. The latest model incorporates channel attention and point attention methods. We will implement this improvement in subsequent experiments to further enhance the model’s performance.

Our research will inspire future studies in camera–radar fusion for 3D object detection.

## Data Availability

The nuScenes dataset is publicly available.
